# Demand satisfied by modern contraceptive among married women of reproductive age in Kenya

**DOI:** 10.1371/journal.pone.0248393

**Published:** 2021-04-09

**Authors:** Peter Gichangi, Michael Waithaka, Mary Thiongo, Alfred Agwanda, Scott Radloff, Amy Tsui, Linea Zimmerman, Marleen Temmerman

**Affiliations:** 1 Technical University of Mombasa, Mombasa, Kenya; 2 Department of Public Health and Primary Care, Faculty of Medicine and Health Sciences, Ghent University, Ghent, Belgium; 3 International Centre for Reproductive Health, Mombasa, Kenya; 4 Population Services Research Institute (PSRI), University of Nairobi, Nairobi, Kenya; 5 Department of Population, Family and Reproductive Health, Johns Hopkins Bloomberg school of public Health, Baltimore, Maryland, United States of America; 6 Aga Khan University, Kenya, Nairobi, Kenya; University of Botswana, BOTSWANA

## Abstract

**Background:**

Demand for family planning met/satisfied with modern contraceptive methods (mDFPS) has been proposed to track progress in Family Planning (FP) programs for Sustainable Development Goals. This study measured mDFPS among married women of reproductive age (MWRA) in Kenya to identify which groups were not being reached by FP programs.

**Materials and methods:**

Performance, Monitoring and Accountability 2020 (PMA2020) survey data from 2014–2018 was used. PMA2020 surveys are cross-sectional including women 15–49 years. PMA2020 used a 2-stage cluster design with urban/rural regions as strata with random selection of households. Univariate and multivariate analysis was done using stata V15.

**Results:**

Of the 34,832 respondents interviewed from 2014 to 2018, 60.2% were MWRA. There was a significant decrease in demand for FP from 2014 to 2018, p = 0.012. Lowest demand was among 15–19 and 45–49 years old women. Overall, modern contraceptive prevalence rate increased significantly from 54.6% to 60.8%, p = 0.004, being higher for women from urban areas, home visits by health care worker (HCW), educated, wealthy, visited health facilities and exposed to mass media. Unmet need for FP decreased from 23.0–13.8% over the 5-years, p<0.001. Married adolescent 15–19 had the highest unmet need and those from rural areas, poor, uneducated and not exposed to mass media. mDFPS increased significantly from 69.7–79.4% over the 5-years, p<0.001, with increase in long acting reversible contraception/permanent methods from 19.9–37.2% and decrease in short acting methods from 49.9–42.2%. Significant determinants of mDFPS were age, rural/urban residence, education, wealth, health facility visitation, exposure to FP messages via mass media in the last 12 months, year of study and county of residence.

**Conclusions:**

Results show a good progress in key FP indicators. However, not all MWRA are being reached and should be reached if Kenya is to achieve the desired universal health coverage as well as Sustainable Development Goals. Targeted home visits by HCW as well increase in mass media coverage could be viable interventions.

## Introduction

Kenya has made tremendous progress in improving access and use of family planning (FP) methods with modern contraceptive prevalence rate (mCPR) of 53.3% among married women of reproductive age (MWRA) in 2014 [1]. Traditionally, mCPR has been used to monitor progress for FP programs. For the Sustainable Development Goals (SDGs), an alternative measure has been proposed—the percent demand for family planning met/satisfied with modern contraceptive methods (mDFPS) [2, 3]. This measure is considered to be a good measure to track progress in FP programs because it is considered to reflect family planning’s aim of supporting individuals’ and couples’ right to choose whether and when to have a child by providing them the means to implement their decisions and promotes voluntarism, informed choice, rights, and equity [2, 3]. The proposed global benchmark for mDFPS has been proposed as 75% by 2030 [2, 3]. The mDFPS has routinely been tracked via demographic and health surveys, though there has been limited discussion on its value in program monitoring in comparison to mCPR [2–6], its importance has not been considered. High levels of mDFPS could be a reflection of provision and delivery of safe, effective and accessible modern contraceptive methods, thereby contributing to improvement of women’s sexual and reproductive health needs [7, 8].

Despite the good progress Kenya has made in provision of FP services with a good national average, mCPR of 53.3% among MWRA, there are large disparities in mDFPS between Counties [1]. Studies have shown lower levels of mDFPS among women who are younger, poorer and living in rural areas [9–11]. Ross [9] study showed that where contraceptive use has reached moderately high levels in the general population, the gap between rich and poor can be quite large ranging from 37 to nearly 50 points for the gaps in the CPR. Kenya has committed to Universal Health Coverage (UHC) which includes access to and use of FP as a human right [12, 13] and to ensure no one is left behind. Efforts to achieve universal mDFPS require the assessment of within-country inequalities and identification of low-coverage population subgroups [1, 9, 10]. With devolution where the unit of program implementation is the county, it is important to understand whether there are groups of MWRA who may be lagging behind in mDFPs at the County level. Our objective was to identify which groups of MWRA are not being reached if any by FP programs at counties participating in Performance, Monitoring and Accountability 2020 (PMA2020) project. Performance, Monitoring and Accountability 2020 was one of the projects designed to monitor key Family Planning 2020 (FP2020) indicators to track commitments made to address the delivery, financing and policy to enable that 120 million additional women and girls have access to modern contraception by 2020. Performance, Monitoring and Accountability 2020 was designed to collect data from women of reproductive age (WRA) in line with demographic and health surveys (DHS) to allow comparability.

## Materials and methods

### Data source

Nationally representative data collected via Performance Monitoring and Accountability 2020 (PMA2020) surveys in Kenya from 2014 to 2018 was used. PMA2020 is a cross-sectional survey using two-stage cluster design, with urban-rural stratum with the enumeration area (primary sampling unit) from which households are randomly selected. PMA2020 survey collects household and has service delivery points data from 11 nationally representative of the 47 counties in Kenya. The surveys are modelled on the insight from Demographic and Health Survey. More information on the design of the surveys has been published elsewhere [14].

### Data management

By using Open Data Kit (ODK), sufficient constraints were included to eliminate data capture errors. Once data was uploaded to the aggregation server, data were regularly reviewed and cleaned and 10% of the households were re-interviewed by the supervisors for quality control.

### Study variables

#### Dependent variables

Demand for FP satisfied with modern methods (mDFPS) among women aged 15–49 years who are currently married or living with a man was assessed as the main dependent variable in this analysis. In addition, we also present analysis on modern contraception use as well as unmet need for family planning (total, for spacing, for limiting).

To compute mDFPS among married women, the numerator included all married women 15–49 years old who were using modern contraceptive methods at the time of the survey while the denominator included all married women of reproductive age (15–49 years old) who had a demand for family planning. Women were considered to have a demand for family planning if they wanted to delay, space or limit childbearing. This includes a woman who: is or her partner is currently using a contraceptive method, has an unmet need for family planning, is currently pregnant or postpartum amenorrhoeic whose current pregnancy or last birth was unwanted or mistimed, is currently sexually active and able to become pregnant who say that they want to delay pregnancy by two or more years or do not know when or whether they want any more children and are not currently using any contraceptive method. Modern contraceptive methods were defined as technological products or medical procedures that affect natural reproduction [15] which include: long acting reversible contraception—IUDs and hormone implants; permanent methods—male and female sterilizations; and short acting methods including injectables, contraceptive pills, condoms, diaphragms, spermicidal agents and emergency contraception. The Lactational Amenorrhea Method (LAM), was included as a short acting modern method in this analysis [15].

#### Independent variables

Analyses in this study were stratified by woman’s age (15–19, 20–24, 25–29, 30–34, 35–39, 40–44, 45–49 years); wealth quintiles, based on the asset index included in the survey datasets (Q1 being the poorest and Q5 the richest quintile); level of education (none; primary; secondary or higher); area of residence (urban or rural); region/ county of residence and year of data collection. Other important covariate considered in this analysis include: visit to a health facility in the last 12 months preceding the survey, discussion about FP at the health facility, being visited by a family planning community health worker in the last 12 months and exposure to family planning messages via mass media in the last 12 months.

### Data analysis

The data were first summarized with percentages. For the summary statistics, the study years (2014 and 2015) where data was collected biannually (June/July and Nov/Dec), data from the two rounds were pooled (i.e. in 2014, round 1 and 2 data were pooled and in 2015, round 3 and 4 data were pooled). The chi-square test of independence for a two-way contingency table was used in the bivariate analysis. But because of the complex sampling design, the corrected weighted Pearson chi-square statistic (Design-based F statistic), was instead conducted to assess the association between the dependent variable of interest and the covariates within and across the five survey years. Trends in mDFPS according to modern contraceptive method type: a) long acting reversible and permanent contraception; b) Short acting methods were also explored.

All the covariates including the survey years were further subjected to multivariable analyses using enter method to identify the significant covariates of mDFPS, modern contraceptives use (mCPR) and unmet need for FP while controlling for the extraneous influence of the survey years using a pooled dataset. A robust Poisson regression model was used for the multivariable analyses to obtain the prevalence ratio (PR) with robust 95% Confidence Intervals (CI).

The complex survey design and weights (individual sampling weights for women) were taken into account during the analysis. STATA 15.0 statistical software was used for all analyses (Stata Corporation, College Station, TX, USA). P ≤ 0.05 was considered significant.

### Ethical considerations

Written informed consent was obtained for all participants 18 and above. For minors, parental/guardian consent and minor assent were obtained. Ethical approval was provided by Kenyatta National Hospital/University of Nairobi ethical review committee (REF: P15/01/2014) and National Commission for Science, Technology and Innovation (REF: NACOSTI/P/14/0813/1676).

### Results

In total, there were 20,956 married women out of the 34,832 women of reproductive age interviewed between the years 2014 and 2018. The analysis was restricted to only married women of reproductive age, 15–49 years, breakdown given in Table 1. Across the first four survey years, majority of the participants were age 25–29 years while in 2018 the majority of the participants were age 30–34 years. About 6 or 7 in every 10 participants were residing in rural area; more than 90% had some formal education and at least 4 in every 10 participants were either from households in the lower/lowest wealth quintile, Table 1.

**Table 1:** Demographic and socio-economic characteristics of respondents.

	PMA2014**	PMA2015**	PMA2016	PMA2017	PMA2018
All women	8,083	9,317	5,885	5,876	5,671
Married women (%)	5148 (63.7)	5570 (59.8)	3497 (59.4)	3404 (57.9)	3337 (58.8)
**Age group**					
15–19	3.2	3.4	3.1	2.9	2.7
20–24	18.9	19.8	18.0	17.8	16.1
25–29	27.2	25.0	23.4	23.3	21.7
30–34	17.7	19.0	20.2	20.7	23.7
35–39	14.6	14.5	14.6	14.7	14.8
40–45	11.0	9.7	11.9	12.1	13.0
45–49	7.3	8.6	8.7	8.4	8.0
**Residence**					
Rural	60.9	62.1	70.4	73.4	71.1
Urban	39.1	37.9	29.6	26.6	28.9
**Education**					
No Education	4.6	4.9	6.4	6.8	5.9
Primary / technical	56.1	55.6	54.8	55.0	53.3
Secondary	26.6	26.3	26.9	25.7	27.5
Higher	12.8	13.3	11.9	12.4	13.2
**Wealth quintile**					
Lowest quintile	26.1	21.4	19.2	20.4	20.2
Lower quintile	22.1	22.4	22.2	23.1	22.3
Middle quintile	17.9	19.5	21.1	21.2	20.3
Higher quintile	16.6	17.8	18.0	17.2	18.0
Highest quintile	17.3	19.0	19.5	18.1	19.1
**County of residence**					
Bungoma	8.9	8.9	8.9	9.7	9.2
Kericho	13.0	12.6	10.4	9.9	9.1
Kiambu	10.5	10.7	6.5	5.5	6.1
Kilifi	11.9	12.4	9.1	9.0	8.4
Kitui	10.3	11.5	8.3	8.5	8.2
Nairobi	17.8	16.7	10.3	9.3	11.4
Nandi	8.8	8.3	8.0	8.7	9.2
Nyamira	8.6	8.4	7.7	7.6	7.3
Siaya	10.1	10.7	7.4	8.0	6.5
Kakamega*	-	-	17.6	17.0	18.1
West Pokot*	-	-	5.8	6.8	6.5

Note:

*Kakamega and West Pokot included in 2016 using same process of County selection;

** In 2014 and 2015, there were two rounds of data collection 6-months apart

From 2014 to 2018, the overall demand for FP ranged between 75.4% and 79.8%. More than three quarters of married women had demand for modern contraception in each year (>75%). The overall demand has significantly decreased since 2014 (p = 0.012). Across the years, the demand for FP was lowest among women age 45–49 years and 15–19 years. The demand for FP among rural women was not significantly different from urban women across all years (p>0.05). In the initial rounds of PMA survey, total demand for family planning was not significantly different among women from the different wealth quintile, among women who had or had not been visited by a community health worker in the last 12 months and those who had or had not visited a health facility in the last 12 months (p>0.05). By 2018, the demand for FP was significantly low among women with no formal education, women from the lowest wealth quintile, those who had not been visited by a community health worker as well as among those had not been exposed to family planning messages via mass media in the last 12 months (p<0.05), Table 2.

**Table 2:** Total demand, met and unmet need for modern contraception.

	Total demand for modern contraception					Modern contraceptive methods use (mCPR)					Total unmet need				
	PMA2014	PMA2015	PMA2016	PMA2017	PMA2018	PMA2014	PMA2015	PMA2016	PMA2017	PMA2018	PMA2014	PMA2015	PMA2016	PMA2017	PMA2018
**Total**	78.2	79.8	76.8	75.4	76.6	54.6	60.7	60.0	59.2	60.8	23.0	17.0	15.2	14.9	13.8
**Age group**															
15–19	66.0	66.2	65.4	65.9	55.3	46.1	36.9	38.2	45.0	30.2	19.7	22.1	27.2	20.9	25.1
20–24	73.7	75.9	77.4	74.8	75.9	49.6	55.6	60.4	58.3	59.8	23.1	19.8	15.9	15.8	14.8
25–29	81.5	84.0	82.2	74.2	78.7	61.2	66.2	65.8	59.7	63.3	20.0	16.1	15.2	13.6	13.0
30–34	82.2	86.1	82.5	82.3	81.3	57.0	68.3	67.2	65.0	65.3	24.4	15.8	13.7	16.1	14.1
35–39	87.4	84.7	81.6	81.0	83.7	62.5	65.1	63.1	65.7	67.3	24.0	17.4	16.6	13.9	14.3
40–45	77.9	81.3	72.4	79.2	75.1	50.0	60.8	52.2	62.0	59.4	27.5	17.2	17.5	14.7	13.9
45–49	55.8	58.4	49.9	51.1	54.9	31.7	41.3	40.0	35.6	43.4	23.3	12.7	7.7	13.4	8.3
**Residence**															
Rural	77.8	79.5	77.1	74.4	76.7	51.6	59.6	58.9	57.3	60.7	25.8	18.5	16.8	16.1	14.2
Urban	78.9	80.2	76.3	78.2	76.4	59.1	62.4	62.7	64.7	61.1	18.7	14.5	11.5	11.6	12.9
**Education**															
No Education	68.5	66.9	50.1	41.9	40.6	26.8	30.5	21.5	19.2	22.5	41.1	33.8	27.8	22.3	17.8
Primary /technical	78.9	80.2	78.8	77.4	78.4	53.5	60.2	61.0	60.2	60.5	24.8	18.5	16.7	16.1	16.4
Secondary	79.9	81.1	79.0	79.8	79.5	60.2	66.0	65.5	65.5	66.9	18.7	12.8	11.6	12.4	10.6
Higher	75.4	80.0	77.6	76.0	79.2	57.2	63.3	63.7	63.9	66.5	17.4	12.9	9.9	10.5	8.5
**Wealth quintile**															
Lowest	79.2	79.0	69.6	65.7	69.8	50.4	58.4	44.7	45.4	48.1	28.3	18.9	24.0	19.6	20.2
Lower	77.9	78.9	80.0	77.0	81.2	53.3	56.8	62.7	58.8	66.9	24.0	21.1	16.8	17.0	12.3
Middle	78.5	80.2	77.9	76.9	77.5	55.3	62.2	63.1	60.6	63.0	22.1	16.7	12.7	14.9	12.7
Higher	77.5	80.5	81.1	81.2	77.5	57.9	65.8	67.0	69.4	63.4	19.3	13.1	12.3	10.4	12.5
Highest	77.6	80.6	75.3	77.0	76.5	58.5	61.4	62.3	64.1	62.4	18.2	13.9	10.3	11.1	11.4
**Visited by FP worker in the last 12 months**															
No	77.9	79.5	76.0	75.1	75.3	53.9	60.4	59.2	58.6	59.4	23.3	16.9	15.2	15.2	14.0
Yes	80.6	82.1	81.3	77.4	83.8	59.6	63.0	64.2	63.6	68.5	20.6	17.3	15.3	12.6	12.9
**Visited health facilities in the last 12 months**															
No	76.4	77.0	72.6	74.5	74.7	51.8	56.1	54.2	59.2	59.7	24.1	18.7	17.1	14.3	13.3
Yes	79.0	80.6	79.2	76.0	77.6	55.7	61.9	63.2	59.2	61.4	22.5	16.5	14.2	15.2	14.1
**Told of FP at the health facility**															
No	77.5	79.6	77.1	73.4	74.5	54.0	61.2	60.3	56.6	58.5	22.5	15.7	14.5	15.0	14.1
Yes	80.9	81.5	81.0	78.4	80.5	57.8	62.6	65.8	61.7	64.1	22.6	17.4	13.8	15.4	14.1
**Exposed to FP messages via mass media in the last 12 months**															
No	73.7	75.4	59.4	60.0	62.5	43.8	51.6	36.0	39.1	42.6	28.9	22.1	22.9	20.3	18.4
Yes	78.9	80.3	78.9	77.6	78.3	56.0	61.8	62.9	62.1	63.1	22.2	16.4	14.3	14.1	13.3
**County of residence**															
Bungoma	82.0	82.0	82.8	80.0	81.0	54.5	61.7	62.7	65.1	64.6	27.1	19.2	19.1	14.4	13.9
Kericho	79.2	81.9	81.3	82.5	75.7	52.0	58.8	64.3	63.6	59.8	26.1	20.9	15.6	15.5	11.6
Kiambu	79.7	80.2	72.8	73.2	74.6	66.1	64.9	60.4	65.4	64.6	13.1	13.5	11.1	7.8	6.7
Kilifi	69.4	71.5	69.1	67.2	66.6	32.4	43.4	42.7	41.1	43.8	36.6	26.8	24.3	24.3	22.6
Kitui	75.3	81.6	78.5	76.5	81.2	53.9	67.1	65.6	60.4	68.2	20.6	13.0	11.5	13.8	10.6
Nairobi	79.3	81.6	75.1	79.2	77.3	59.2	61.3	59.5	65.3	60.6	19.6	14.8	13.1	12.5	14.6
Nandi	85.1	80.6	79.8	79.8	82.5	59.8	66.9	65.2	60.5	66.4	24.4	12.8	12.5	17.2	15.1
Nyamira	80.0	82.0	82.5	77.8	73.5	64.7	72.1	71.5	66.2	64.0	13.7	8.2	9.7	10.9	8.5
Siaya	76.2	77.7	76.6	75.5	74.4	51.2	56.3	56.6	56.6	57.7	24.8	20.4	18.7	18.2	15.5
Kakamega*	-	-	82.1	82.5	87.9	-	-	67.8	70.6	73.6	-	-	12.9	11.2	12.6
West Pokot*	-	-	50.0	38.4	45.1	-	-	25.9	19.2	22.0	-	-	22.2	19.2	20.8

Note:

*Kakamega and West Pokot were added in 2016 using the same procedure for selection of the initial counties, PPS based on number of households; Total Demand for Modern Contraception = Met Demand+Unmet Need, where Met Demand = Total Modern Contraceptive Use

Since 2014, modern contraceptives use among married women significantly increased from 54.6% to 60.8% in 2018 (p = 0.004). Across the years, modern contraceptives use was highest among married women between age 30–34 and 35–39 years, among women in urban setup, women with some formal education, women from the high wealth quintiles, among women who had visited a health facility in the last 12 months and discussed family planning, among women who had been visited by a health worker in the last 12 months and among those who had been exposed to family planning messages via mass media in the last 12 months. Among the different counties, Nyamira had the highest rate of modern contraception use in 2014 through 2016 while Kakamega had the highest rate of modern contraception use in 2017 and 2018. Whereas all other counties recorded an increase in modern contraceptives use between 2014 and 2018, Kiambu, Nyamira and West Pokot counties recorded a decrease.

The unmet need for family planning significantly decreased from 23.0% in 2014 to 13.8% in 2018 among MWRA (p<0.001). In 2015 through 2018, total unmet need was highest among adolescents age 15–19 years. Total unmet need for FP had been consistently higher among married women in rural areas, among women with no formal education, among women from households in the lowest wealth quintile, among those who have not been exposed to family planning messages via mass media in the last 12 months. Kilifi County had consistently had higher total unmet need for family planning over the five study years than other counties.

There has been a significant increase (p<0.001) in mDFPS from 69.7% in 2014 to 79.4% in 2018, Fig 1. A similar trend is seen for mDFPS satisfied by long acting reversible and permanent contraceptive methods increasing from 19.6% in 2014 to 2018, 36.9%, (p<0.001). Conversely, demand satisfaction by short acting modern contraceptive methods have significantly decreased from 49.9% in 2014 to 42.2% in 2018 (p = 0.006).

**Fig 1:**
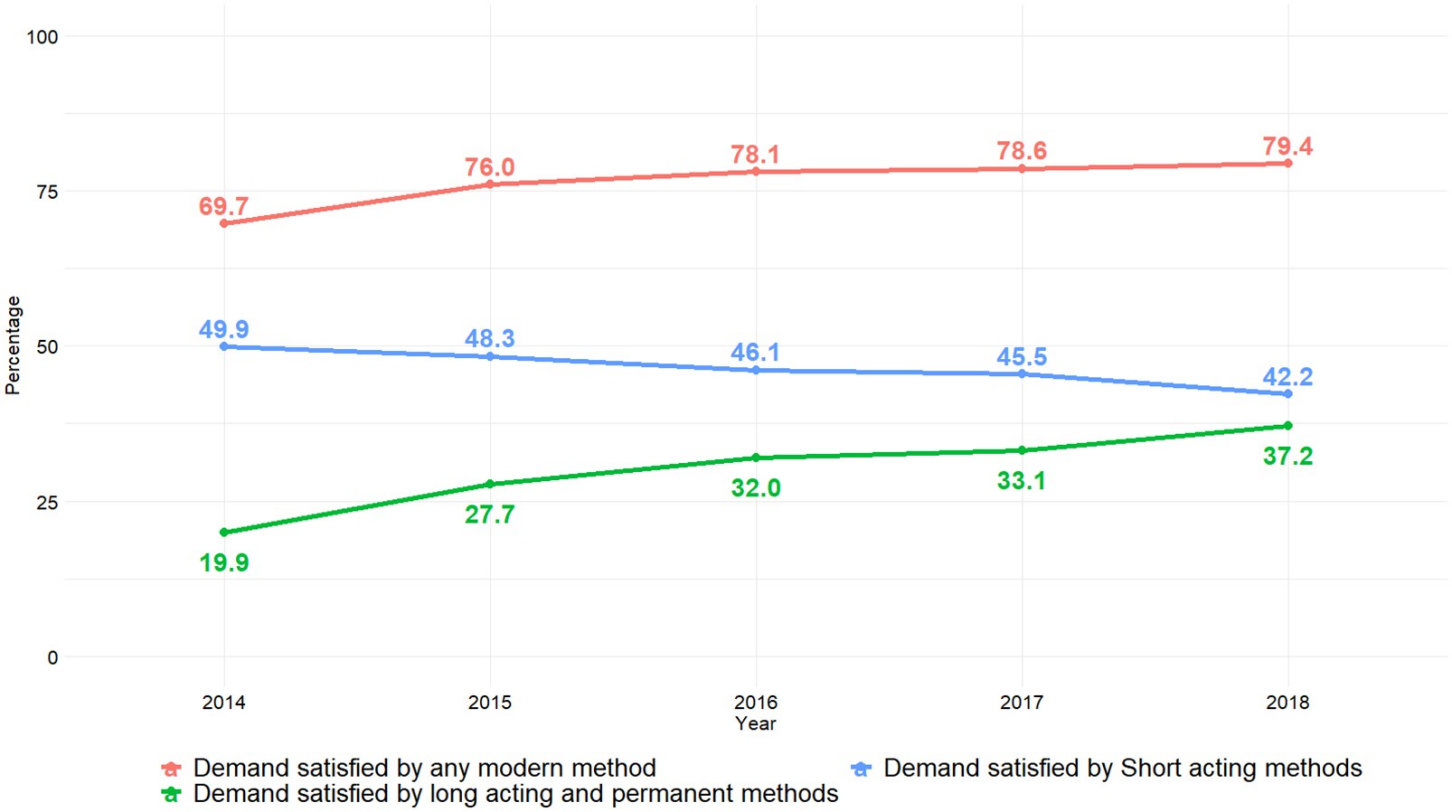
Demand for family planning satisfied by modern methods.

Design-based F-test results in Table 3, shows that the respondents’ level of education, exposure to FP messages via mass media in the last 12 months, household wealth and county of residence are the factors significantly associated with mDFPS consistently in each of the five study years (p<0.05). In addition, the respondents’ age has significant association with mDFPS except for the year 2017. Residence of the respondents had a significant association with mDFPS consistently in the years 2014, 2016 and 2017. To examine if there were changes in mDFPS within the subgroups of the study population across the study years, significance tests were also applied for each population subgroups across the years.

**Table 3:** Demand for family planning satisfied by modern contraceptive methods disaggregated by the selected characteristics across the years.

Variable	PMA2014	PMA2015	PMA2016	PMA2017	PMA2018	P-value**
**Age group**	**P**^**1**^ **= 0.002**	**P**^**1**^ **= 0.002**	**P**^**1**^ **= 0.001**	P^1^ = 0.098	**P**^**1**^ **= 0.017**	
15–19	69.77	55.69	58.45	68.35	54.56	P^2^ = 0.299
20–24	67.28	73.35	78.01	78	78.75	**P**^**2**^ **= 0.002**
25–29	75.01	78.78	80.03	80.46	80.52	P^2^ = 0.173
30–34	69.41	79.39	81.48	78.91	80.36	**P**^**2**^**<0.001**
35–39	71.45	76.91	77.29	81.11	80.36	**P**^**2**^ **= 0.023**
40–45	64.18	74.78	72.08	78.3	79.11	**P**^**2**^ **= 0.001**
45–49	56.79	70.71	80.06	69.63	79.02	**P**^**2**^ **= 0.003**
**Residence**	**P**^**1**^**<0.001**	P^1^ = 0.236	**P**^**1**^ **= 0.025**	**P**^**1**^ **= 0.008**	P^1^ = 0.701	
Rural	66.35	74.97	76.38	76.99	79.15	**P**^**2**^**<0.001**
Urban	74.91	77.78	82.18	82.74	80.01	**P**^**2**^ **= 0.005**
**Education**	**P**^**1**^**<0.001**	**P**^**1**^**<0.001**	**P**^**1**^**<0.001**	**P**^**1**^**<0.001**	**P**^**1**^**<0.001**	
No Education	39.11	45.63	42.95	45.79	55.33	P^2^ = 0.504
Primary / technical	67.84	75	77.45	77.84	77.18	**P**^**2**^**<0.001**
Secondary	75.39	81.36	82.93	82.15	84.12	**P**^**2**^ **= 0.002**
Higher	75.87	79.11	82.14	84.06	83.94	P^2^ = 0.110
**Wealth quintile**	**P**^**1**^**<0.001**	**P**^**1**^ **= 0.024**	**P**^**1**^**<0.001**	**P**^**1**^**<0.001**	**P**^**1**^**<0.001**	
Lowest quintile	63.55	74.02	64.16	69.15	68.87	**P**^**2**^ **= 0.022**
Lower quintile	68.38	71.94	78.37	76.35	82.42	**P**^**2**^ **= 0.001**
Middle quintile	70.49	77.56	80.96	78.86	81.23	**P**^**2**^ **= 0.010**
Higher quintile	74.72	81.66	82.6	85.43	81.79	**P**^**2**^ **= 0.001**
Highest quintile	75.45	76.2	82.73	83.26	81.53	**P**^**2**^ **= 0.015**
**Visited by FP worker in the last 12 months**	P^1^ = 0.066	P^1^ = 0.735	P^1^ = 0.714	P^1^ = 0.154	P^1^ = 0.242	
No	69.15	75.94	77.9	78.03	78.92	**P**^**2**^**<0.001**
Yes	73.86	76.74	78.97	82.26	81.79	P^2^ = 0.102
**Visited health facilities in the last 12 months**	P^1^ = 0.310	P^1^ = 0.079	P^1^ = 0.091	P^1^ = 0.458	P^1^ = 0.712	
No	67.77	72.89	74.68	79.49	79.93	**P**^**2**^ **= 0.001**
Yes	70.51	76.87	79.81	77.99	79.14	**P**^**2**^**<0.001**
**Told of FP at the health facility**	P^1^ = 0.455	P^1^ = 0.978	P^1^ = 0.209	P^1^ = 0.564	P^1^ = 0.582	
No	69.75	76.86	78.16	77.12	78.49	**P**^**2**^ **= 0.002**
Yes	71.44	76.81	81.21	78.75	79.67	**P**^**2**^ **= 0.001**
**Exposed to FP messages via mass media in the last 12 months**	**P**^**1**^ **= 0.001**	**P**^**1**^ **= 0.004**	**P**^**1**^**<0.001**	**P**^**1**^**<0.001**	**P**^**1**^ **= 0.002**	
No	59.38	68.43	60.58	65.15	68.25	P^2^ = 0.297
Yes	71.04	76.89	79.65	80.06	80.5	**P**^**2**^**<0.001**
**County of residence**	**P**^**1**^**<0.001**	**P**^**1**^ **= 0.001**	**P**^**1**^ **= 0.001**	**P**^**1**^**<0.001**	**P**^**1**^**<0.001**	
Bungoma	66.52	75.23	75.71	81.46	79.77	**P**^**2**^ **= 0.006**
Kericho	65.72	71.76	79.08	77.09	79.04	P^2^ = 0.083
Kiambu	82.9	81.01	83.07	89.37	86.66	P^2^ = 0.499
Kilifi	46.66	60.64	61.74	61.21	65.71	**P**^**2**^ **= 0.018**
Kitui	71.63	82.27	83.59	78.95	84.1	P^2^ = 0.183
Nairobi	74.62	75.13	79.32	82.41	78.34	P^2^ = 0.175
Nandi	70.22	83.02	81.62	75.78	80.46	P^2^ = 0.105
Nyamira	80.91	87.89	86.67	85.08	87.08	P^2^ = 0.231
Siaya	67.13	72.43	73.83	74.89	77.52	P^2^ = 0.081
Kakamega*	-	-	82.64	85.6	83.81	P^2^ = 0.710
West Pokot*	-	-	51.84	50.07	48.85	P^2^ = 0.938

Note:

*Kakamega and West Pokot were added in 2016;

** Design-based F-statistic;

p^1^ –P-value for test of significance of the covariates within each year of study; p^2^- P-value for test of significance of the change in the mDFPS in each subgroup across the study years.

Demand for FP satisfied with modern methods significantly increased (p<0.05) across the years among women in both rural and urban residence, women from all wealth quintiles, women who had visited a health facility in the last 12 months as well as those who didn’t visit, women who had been told about FP at the health facility during their visit as well as women who were never told of FP at health facilities. Similarly, in majority of the subgroups of the study population, the mDFPS significantly increased (p<0.05) across the years, with exception of married adolescents (15–19 years) and those age 25–29 years; women with no formal education and those with higher education; women who had been visited by a family planning community health worker in the last 12 months; as well as women who had not been exposed to FP messages via mass media in the last few months. Bungoma and Kilifi counties registered significant increase in mDFPS (p<0.05). West Pokot county registered a nonsignificant decrease in mDFPS between 2016 and 2018. Increment of mDFPS in other counties were not statistically significantly different (p>0.05), Table 3.

Table 4 shows results from the multivariable analysis for covariates of mDFPS, modern contraceptives use and unmet need for FP after adjusting for the influence of year of data collection and county of residence. The demand for family planning satisfied by modern methods in 2015, 2016, 2017 and 2018 were significantly higher than in 2014 (p<0.001). After controlling for the influence of the years of study and other covariates, the results show that married women aged 20–24, 25–29, 30–34, 35–39 and 40–44 were significantly more likely to have their demand for family planning satisfied by modern methods compared to those age 15–19 years (adolescents) (p<0.05); whereas demand satisfaction by modern methods among married women age 45–49 was not significantly different compared to the adolescent women. Married women residing in the urban areas were more likely to have their demand for family planning satisfied by modern methods than those who reside in rural areas. Married women with some form of formal education were more likely to have their demand for family planning satisfied by modern methods than those with no formal education (p<0.001). Married women in the lower to the highest wealth quintiles were more likely to have their demand for family planning satisfied by modern methods as compared to married women in lowest wealth quintiles. Married women who had visited health facilities or had been exposed to FP messages via mass media in the last 12 months were more likely to have their demand for family planning satisfied by modern methods as compared to married women who had not. Comparing demand of family planning satisfaction by modern methods among married women from each of the study counties with married women from Nyamira County, we observed that married women in all other counties had significantly lower likelihood to have their demand satisfied by modern methods than married women in Nyamira county (p<0.05). Similar observations are observed for modern contraceptive use among married women in the reproductive age.

**Table 4:** Multivariable analysis for mDFPS, mCPR and unmet need for FP.

Variable	mDFPS		mCPR		Unmet need	
	Adjusted PR (95% CI)	Wald tests P-value	Adjusted PR (95% CI)	Wald tests P-value	Adjusted PR (95% CI)	Wald tests P-value
**Age group**						
15–19	Ref.	**<0.001**	Ref.	**<0.001**	Ref.	**<0.001**
20–24	1.17 (1.07, 1.29)**		1.33 (1.19, 1.5)**		0.92 (0.77, 1.1)	
25–29	1.24 (1.13, 1.36)**		1.51 (1.35, 1.69)**		0.8 (0.67, 0.96)*	
30–34	1.22 (1.11, 1.34)**		1.54 (1.38, 1.72)**		0.87 (0.72, 1.04)	
35–39	1.21 (1.11, 1.33)**		1.54 (1.38, 1.73)**		0.89 (0.74, 1.07)	
40–44	1.14 (1.04, 1.25)**		1.34 (1.2, 1.51)**		0.95 (0.78, 1.15)	
45–49	1.09 (0.99, 1.21)		0.92 (0.81, 1.04)		0.67 (0.54, 0.82)**	
**Residence**						
Rural	Ref.	**0.038**	Ref.	0.197	Ref.	**0.020**
Urban	1.03 (1, 1.05)*		1.02 (0.99, 1.06)		0.89 (0.81, 0.98)*	
**Education**						
No Education	Ref.	**<0.001**	Ref.	**<0.001**	Ref.	**<0.001**
Primary / technical	1.42 (1.28, 1.58)**		1.74 (1.53, 1.97)**		0.82 (0.71, 0.93)**	
Secondary	1.48 (1.33, 1.65)**		1.81 (1.59, 2.06)**		0.67 (0.57, 0.78)**	
Higher	1.45 (1.3, 1.62)**		1.7 (1.49, 1.95)**		0.64 (0.53, 0.78)**	
**Wealth quintile**						
Lowest	Ref.	**<0.001**	Ref.	**<0.001**	Ref.	**0.001**
Lower	1.06 (1.02, 1.09)**		1.07 (1.03, 1.11)**		0.91 (0.83, 1)*	
Middle	1.07 (1.03, 1.11)**		1.07 (1.02, 1.11)**		0.85 (0.77, 0.95)**	
Higher	1.11 (1.07, 1.15)**		1.13 (1.08, 1.18)**		0.77 (0.67, 0.88)**	
Highest	1.09 (1.05, 1.14)**		1.08 (1.02, 1.15)**		0.75 (0.64, 0.89)**	
**Visited health facilities in the last 12 months**						
No	Ref.	**0.013**	Ref.	**0.002**	Ref.	0.075
Yes	1.03 (1.01, 1.05)*		1.05 (1.02, 1.08)**		0.93 (0.87, 1.01)	
**Exposed to FP messages via mass media in the last 12 months**						
No	Ref.	**<0.001**	Ref.	**<0.001**	Ref.	0.247
Yes	1.08 (1.03, 1.12)**		1.14 (1.08, 1.2)**		0.95 (0.86, 1.04)	
**Year**						
2014	Ref.	**<0.001**	Ref.	**<0.001**	Ref.	**<0.001**
2015	1.08 (1.05, 1.12)**		1.11 (1.07, 1.16)**		0.75 (0.68, 0.83)**	
2016	1.1 (1.07, 1.14)**		1.09 (1.05, 1.14)**		0.69 (0.62, 0.77)**	
2017	1.11 (1.08, 1.15)**		1.09 (1.04, 1.13)**		0.66 (0.59, 0.74)**	
2018	1.12 (1.08, 1.15)**		1.1 (1.05, 1.15)**		0.63 (0.56, 0.7)**	
**County**^£^						
Nyamira	Ref.	**<0.001**	Ref.	**<0.001**	Ref.	**<0.001**
Bungoma	0.88 (0.85, 0.91)**		0.9 (0.86, 0.94)**		1.85 (1.59, 2.16)**	
Kericho	0.84 (0.81, 0.88)**		0.85 (0.81, 0.89)**		1.91 (1.62, 2.25)**	
Kiambu	0.93 (0.89, 0.97)**		0.9 (0.85, 0.96)**		1.38 (1.11, 1.71)**	
Kilifi	0.73 (0.7, 0.77)**		0.68 (0.63, 0.72)**		2.4 (2.05, 2.8)**	
Kitui	0.92 (0.89, 0.96)**		0.92 (0.87, 0.96)**		1.42 (1.2, 1.69)**	
Nairobi	0.84 (0.8, 0.88)**		0.84 (0.79, 0.89)**		2.02 (1.65, 2.48)**	
Nandi	0.91 (0.88, 0.94)**		0.93 (0.89, 0.97)**		1.64 (1.39, 1.93)**	
Siaya	0.85 (0.82, 0.89)**		0.82 (0.78, 0.86)**		1.86 (1.58, 2.18)**	
Kakamega	0.96 (0.92, 1)*		1.03 (0.98, 1.09)		1.35 (1.08, 1.69)**	
West Pokot	0.62 (0.56, 0.69)**		0.42 (0.37, 0.48)**		1.83 (1.48, 2.25)**	

Note:

^£^- PR and 95% CI are sensitive to the county considered to be reference; Nyamira was chosen to be the reference county because it is one of the counties with the highest mDFPS,

**- Significant at 1% level of significance (los);

*- Significant at 5% los;

CI-Confidence Interval; PR-Prevalence ratio; Ref-Reference category

From the results we observe that after adjusting for the other covariates, the respondents’ age, residence (rural/urban), education, wealth and county of residence are the significant covariates of the total unmet need for FP. Indeed women age 25–29 and 45–49 have significantly lower likelihood of having an unmet need for FP compared to women age 15–19. Also, women with some form of formal education (primary, secondary/ tertiary and higher education) are less likely to have unmet need for FP compared to women with no education. Similarly, women in the lower, middle, higher or the highest wealth quintile are less likely to have unmet need for FP compared to women with in the lowest wealth quintile. By county, women residing in all other counties have significantly higher likelihood of having unmet need for FP as compared to women living in Nyamira county, Table 4.

## Discussion

Kenya has made great strides in provision of FP services for its population. Modern contraceptives use among MWRA significantly increased from 54.6% in 2014 to 60.8% in 2018 (p = 0.004). There was a significant decrease in unmet need for FP from 23.0% in 2014 to 13.8% in 2018 among MWRA (p<0.001). However, total demand for FP among MWRA has decreased from 78.2% in 2014 to 76.6% in 2018 (p = 0.012).

Demand satisfied by modern contraceptives (mDFPS) is a coverage of interventions indicator among the 100 core health indicators by WHO [16]. Trend analysis for demand mDFPS shows there has been a significant increase from 69.7% in 2014 to 79.4% in 2018 (p<0.001). Our findings are similar to those of Hellwig et al [17] who showed growth in mDFPS of 1.5% for Eastern and South African countries. Notable is the finding that mDFPS is being accounted for by long-acting methods with a decrease in short acting methods. Total mDFPS is considered to be reflective of the totality of the FP programs [2, 3]. Hellwig et al [17] used mDFPS as the main indicator to evaluate trends in FP in Low and Middle Income Countries. To access equality for Sustainable Development Goals (SDGs), mDFPS has been proposed as the indicator to use with a benchmark set at 75% [2, 5]. Our study shows that Kenya has achieved this benchmark. Goodkind et al [18] study showed how meeting mDFPS would result in substantial benefits to developing countries such as 20% decline in youth dependency. Indeed, there is global consensus that reproductive health and rights are essential in addition to improving reproductive health outcomes and achieving broader improvements in education, economic status and health [18, 19, 21].

Our data shows disparities with low mDFPS being found among married adolescents, MWRA with no education, those from the poorest wealth quintile, Kilifi and West Pokot counties. These findings are similar to those reported by Ewerling et al [20] and Wulifan et al [11]. These sub-groups need to be reached to ensure there are no disparities in use of FP services. In our study, significant correlates of mDFPS were the calendar year of data collection, age, education attainment, wealth, area of residence, health facility visitation, receiving FP message from media and county of residence. Similar findings were reported by Ewerling et al [20] from a study including Demographic and Health Survey and Mixed Indicator Cluster Surveys data from 77 countries.

To address disparities like shown in our study, the following has been suggested by several researchers: advancement in human rights for all regardless of age, sex, marital status, and health, women’s education, empowerment, gender equality, human capital development, reduction of maternal and child mortality as well as the prevention of HIV transmission [21–25]. Fagan et al [25] suggests that leveraging Universal Health Coverage (UHC)-oriented schemes can sustain and further increase FP progress. Under UHC, Fagan et al [25] noted that for this to succeed, governments need to take deliberate steps to (1) target poor and informal sector populations, (2) include family planning in benefits packages, (3) ensure sufficient financing for family planning, and (4) reduce nonfinancial barriers to access. Kenya is in the process of rolling out UHC program and FP is being incorporated in the National Hospital Health Insurance Fund [26].

Our data shows significant increase in mCPR. This is consistent with Cahil et al [4] findings of rapid increase in mCPR relative to what was expected in 2012 in Kenya. Cahil et al [4] reported an average increase of 2.7 percentage points (mCPR of 15.7% (95% UI 4.6–23.0) from 2012 to 2017) while we report average of 1.2 percentage points (54.6% in 2014 to 60.8% in 2018). The Kenya devolved health system could contribute to the progress observed in this study. Qualitative study by McCollum et al [27] found that devolution in Kenya has focused on improving supply side of health services by expanding the availability, geographic and financial accessibility of health services. Tsofa et al [28] found that the implementation of devolution created an opportunity for local level prioritization and community involvement in health sector planning and budgeting hence increasing opportunities for equity in local level resource allocation.

The decrease in demand for FP could be related to the negative consequences of the health care providers strikes in 2016 and prolonged electioneering in 2017. Health care workers strikes have been shown to be quite disruptive in provision of health services in developing [29–31] and developed countries [31–33]. The Kenya FP program shows some resilience given that even though demand was decreasing, unmet need significantly decreased from 2014 to 2018. Meeting the unmet need for family planning has been suggested as part of the strategies to build resilience for communities [34–37].

In interpreting the results of this study, some limitations need to be taken into consideration. Pooled data from five-rounds of survey were included. Necessary statistical adjustment including *svyset* command in Stata designating the round number as a stratum were used to take care of clustering by round since the women included were not independently sampled. Since only married women were included in the study, the results may not be generalizable to all women of reproductive age in Kenya [2, 38]. Another limitation is the reliance on reported information. Reported information suffers from bias due to social desirability response [39] or just misreporting [40]. The use of questions from DHS which have been validated and are extensively used in FP programs mitigated this limitation.

## Conclusions

Acknowledging these limitations, this study shows increase in mCPR and mDFPS and decreasing demand for FP. There are however subgroups including married women who are young, those without formal education and those from poorest wealth quintile who are still lagging behind and not being reached with FP services. There are counties which are also not making significant improvement in mDFPS over time. Efforts to increase FP coverage must be prioritized to subgroups not currently being reached under UHC or other interventions.

## Supporting information

S1 Data (DTA)

## Abbreviations

CI: Confidence Intervals
CPR: Contraceptive Prevalence Rate
DHS: Demographic and Health Survey
FP: Family Planning
FPET: Family Planning Estimation Tool
IUD: Intrauterine Device
KNBS: Kenya National Bureau of Statistics
LAM: Locational Amenorrhea Method
mCPR: Modern Contraceptive Prevalence Rate
mDFPS: Demand for family planning satisfied with modern methods
MICS: Multiple Indicator Cluster Surveys
MWRA: Married Women of Reproductive Age
ODK: Open Data Kit
PMA2020: Performance Monitoring and Accountability 2020
UHC: Universal Health Coverage
